# Incidental pulmonary arteriovenous malformation

**DOI:** 10.1002/jhm.70259

**Published:** 2026-02-23

**Authors:** Mallory A. Von Lotten, Eric Howell, J. Andrew Watson

**Affiliations:** ^1^ Department of Medicine University of Alabama at Birmingham Heersink School of Medicine Birmingham Alabama USA; ^2^ Department of Radiology University of Alabama at Birmingham Birmingham Alabama USA; ^3^ Department of Pediatrics, Division of Hospital Medicine University of Alabama at Birmingham Birmingham Alabama USA

## CASE PRESENTATION

**Figure 1:**
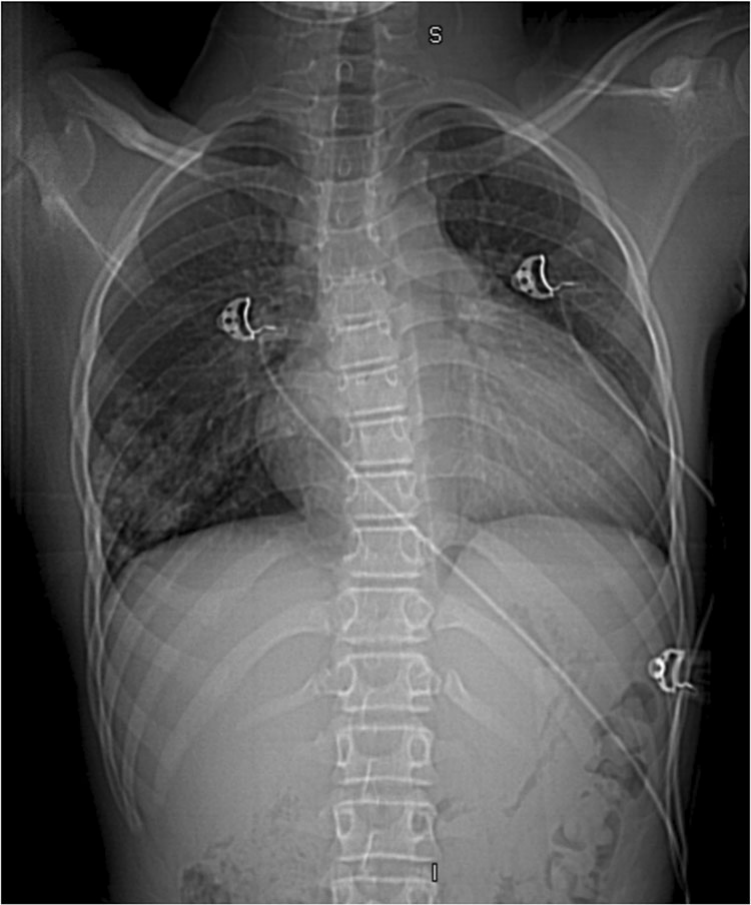
Chest X‐ray of a pediatric pulmonary arteriovenous malformation (AVM). Chest X‐ray demonstrating a well‐circumscribed right lower lobe opacity, prompting further evaluation with advanced imaging.

**Figure 2:**
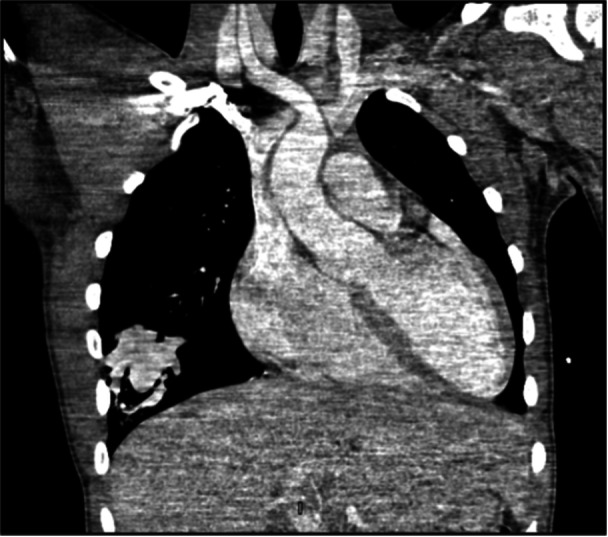
Computed tomography (CT) angiography confirming pulmonary AVM. CT angiography (CTA) revealing a pulmonary arteriovenous malformation (AVM) in the right lower lobe, characterized by a dilated feeding artery and early venous return, explaining the patient′s positional hypoxia.

A 13‐year‐old male presented after a traumatic fall, sustaining left upper extremity fractures. He was hypoxic (80%–90% on room air), requiring supplemental oxygen. Chest X‐ray revealed a patchy right lower lobe opacity (Figure 1), prompting concern for pulmonary pathology. Computed tomography angiography (CTA) demonstrated a pulmonary arteriovenous malformation (AVM) with multiple engorged vessels draining into the right inferior pulmonary vein (Figure 2). The patient reported mild exertional dyspnea but denied chest pain, cyanosis, or syncope. Polycythemia (Hb 17.2 g/dL, Hct 51.2%) suggested chronic hypoxemia. Echocardiography confirmed right‐to‐left shunting, and genetic testing for hereditary hemorrhagic telangiectasia (HHT) was negative. The AVM remains under active surveillance.

## DISCUSSION

This case highlights the incidental discovery of a pulmonary AVM in a pediatric trauma patient, adding complexity to an otherwise trauma‐focused clinical course. Though rare in children, pulmonary AVMs pose significant risks, including sedation‐related complications, pulmonary hemorrhage, ischemic stroke, and heart failure. Chronic extra‐cardiac AVMs can trigger compensatory mechanisms such as erythrocytosis, pulmonary vasodilation, and increased cardiac output, as seen in this patient. The unexpected finding necessitated a multidisciplinary approach involving cardiology, pulmonology, and genetics for safe management and evaluation of hereditary factors.

The maternal history of cyanosis raised suspicion for HHT, an autosomal dominant disorder associated with AVMs in multiple organs.^1^ Early HHT identification is critical, as undiagnosed AVMs can lead to life‐threatening complications. Systematic screening for HHT allows for early AVM detection, risk stratification, and preventive care, underscoring the importance of maintaining a broad differential in pediatric hypoxia cases.

## CONFLICT OF INTEREST STATEMENT

The authors declare no conflicts of interest.

## ETHICS STATEMENT

The authors obtained verbal informed consent, witnessed and documented in accordance with institutional guidelines, for publication of medical information and images.

## Supporting information

supporting information.video 1. axial ct chest view showing a serpiginous vascular structure in the right lung, consistent with a pulmonary arteriovenous malformation (avm).

supporting information.video 2. coronal (or transverse) ct chest view further demonstrating the avm within the right pulmonary vasculature.

## References

[1] Shovlin CL , Guttmacher AE , Buscarini E , et al. Diagnostic criteria for hereditary hemorrhagic telangiectasia (Rendu‐Osler‐Weber syndrome). Am J Med Genet. 2000;91(1):66‐67. 10751092 10.1002/(sici)1096-8628(20000306)91:1<66::aid-ajmg12>3.0.co;2-p

